# Miscellany

**Published:** 1888-07

**Authors:** 


### Miscellany.

At the last meeting of the Ophthalmological and Otological Section of the New York Academy of Medicine, the following motion was made and carried:

"That a committee be appointed, of which the Chairman of the section, Dr. David Webster, be a member, whose duty it shall be to obtain a good photograph of the late Dr. Cornelius R. Agnew, for the purpose of having engravings suitable for framing made from this. The right of issue and sale of such engravings shall be given to some first-class publisher, if practicable; if not, the committee shall offer them to the profession at cost."

In accordance with the above, a committee has been appointed. Members of the profession who desire such an engraving, accompanied by an autograph signature, should send their names and addresses to the secretary of the committee, Dr. Charles H. May, 640 Madison avenue, New York city, at once. When all such names shall have been recorded, those who have requested a copy of the engraving will be notified of the cost of the same, either by the publisher or by the committee having the matter in charge.

---

Medical Aphorisms.—A correspondent signing himself "Artz," sends to the *Canada Lancet* the following professional aphorisms of Amedee Latour:

"1. Life is short, patients fastidious, and the brethren deceptive. 2. Practice is a field, of which tact is the manure. 3. Patients are comparable to flannel—neither can be quitted without danger. 4. The physician who absents himself runs the risk as the lover who leaves his mistress; he is pretty sure to find himself supplanted. 5. Would you rid yourself of a tiresome patient, present your bill. 6. The patient who pays his attendant, is but exacting; he who does not, is a despot. 7. The physician who depends on the gratitude of his patient for his fee, is like the traveler who waited on the bank of a river until it finished flowing, so that he might cross to the other side. 8. Modesty, simplicity, truthfulness!—cleansing virtues, everywhere but at the bedside; there simplicity is construed as *hesitation*, modesty as *want of confidence*, truth as *impoliteness*. 9. To keep within the limits of a dignified assurance without falling into the ridiculous vauntings of the boaster, constitutes the supreme talent of the physician. 10. Remember always to appear to be doing something—above all, when you are doing nothing. 11. With equal, and even inferior, talent, the cleanly and genteely-dressed physician has a great advantage over the untidy one.

---

Pharmacy in the Sixteenth Century.—In the sixteenth century, in England, Boleyn (Queen Anne Boleyn's cousin), a prominent apothecary, laid down the following rules for the practice of pharmacy: The apothecary must first serve God, foresee the end, be cleanly, and pity the poor. His place of dwelling and shop must be cleanly to please the senses withal. His garden must be at hand with plenty of herbs, seeds and roots. He must read Dioscorides. He must have his mortars, stills, pots, filters, glasses, boxes clean and sweet. He must have two places in his shop, one most clean for physic, and the base place for chirurgic stuff. He is neither to decrease nor diminish the physician's prescription. He is neither to buy nor sell rotten drugs. He must be able to open well a vein, for to help pleurisy. He is to meddle only in his vocation, and to remember that his office is to be only the physician's cook.

---

Dr. George M. Sternberg, commissioned by the United States Government to investigate the methods of inoculation practiced in Brazil and Mexico for protection against yellow fever, reports that, so far, no discoveries of practical interest have been made as to the etiology of the disease. During the coming summer, he hopes to pursue his investigations in Havana, making a diligent search for the yellow-fever microbe.

---

Is Consumption Contagious?—After the study of nearly 12,000 cases, Dr. Hermann Brehmer, an able German physician, rejects the theory of the contagiousness of pulmonary consumption. He finds the disease to be due to deficient nutrition of the lung, which may result from many causes. He believes that the operation of all the causes may produce such changes that it may be possible, years in advance, to predict with great probability which members of a family will be afflicted with pulmonary consumption, and which will remain healthy.—*Medical Herald.*

If antipyrin be dissolved in sweet spirits of nitre, the resulting solution soon assumes a rich green color, and, what is more remarkable, a new compound, possessing highly poisonous qualities, is formed. Both of these drugs being recommended for fever, it is not unnatural for the advocate of polypharmacy to combine the two in the same prescription, trusting thereby to obtain an antipyretic of greater value than either alone. This has been done in one case, and—the patient died.

---

Dr. H. C. Yarrow is now publishing, in *Forest and Stream*, an interesting series of articles on "Snake Bite and its Antidote." Dr. Yarrow is Curator of Department of Reptiles, U. S. National Museum, and has abundant facilities for the study of this subject. We would suggest that these articles be collected and published as a monograph, as we have not seen before so exhaustive and scientific an investigation of the subject.

---

There have been, during the past month, in the Sisters of Charity Hospital, two cases of tetanus, following compound comminuted fractures. Both of these cases passed through the Emergency Hospital, a fact giving some color to the theory of the infectious nature of this disease.

---

Dr. O'Hanlan, of the Gouverneur Hospital, reports a case of œdema of the lungs, which was relieved by turning the patient flat on the chest, with the head hanging down. The serum flowed from the mouth and nose, and the breathing was promptly relieved.—*New York Medical Times.*

---

Honors to von Brucke.—The Emperor of Austria has conferred on Hofrath Professor E. Brücke, Professor of Physiology in the University of Vienna, the Order of the "Ehrenzeichen für Kunst und Wissenschaft." This distinction was created by the Emperor last year, and has hitherto, as far as medical men are concerned, been conferred only on Hyrtl.

---

The Suppression of Quack Medicines in Germany.—Kultusminister von Gossler recently announced his intention of securing the coöperation of the Imperial Government in suppressing all advertisements of secret remedies throughout the German States.

